# Measuring Concurrency Attitudes: Development and Validation of a Vignette-Based Scale

**DOI:** 10.1371/journal.pone.0163947

**Published:** 2016-10-20

**Authors:** Anna B. Cope, Catalina Ramirez, Robert F. DeVellis, Robert Agans, Victor J. Schoenbach, Adaora A. Adimora

**Affiliations:** 1 Division of Infectious Diseases, School of Medicine, University of North Carolina at Chapel Hill, Chapel Hill, North Carolina, United States of America; 2 Department of Health Behavior, University of North Carolina Gillings School of Global Public Health, Chapel Hill, North Carolina, United States of America; 3 Department of Biostatistics, University of North Carolina Gillings School of Global Public Health, Chapel Hill, North Carolina, United States of America; 4 Department of Epidemiology, University of North Carolina Gillings School of Global Public Health, Chapel Hill, North Carolina, United States of America; Emory University School of Public Health, UNITED STATES

## Abstract

**Background:**

Concurrent sexual partnerships (partnerships that overlap in time) may contribute to higher rates of HIV transmission in African Americans. Attitudes toward a behavior constitute an important component of most models of health-related behavior and behavioral change. We have developed a scale, employing realistic vignettes that appear to reliably measure attitudes about concurrency in young African American adults.

**Methods:**

Vignette-based items to assess attitudes about concurrency were developed following focus groups and cognitive testing of items adapted from existing scales assessing psychosocial constructs surrounding related sexual behaviors. The new items were included in a telephone survey of African American adults (18–34 years old) in Eastern North Carolina immediately before and after a radio campaign designed to discourage concurrency. We performed an exploratory factor analysis on each sample (pre- and post-campaign) to cross-validate results. We retained factors with a primary loading of ≥0.50 and no secondary loading >0.30. Cronbach’s coefficient alpha was used to evaluate internal reliability. Associations in the predicted direction between the mean responses to items on the final factor and known correlates of concurrency validated the scale.

**Results:**

Factor analysis in a random pre-campaign subsample yielded a one-factor 6-item scale with acceptable internal consistency (Cronbach’s α = 0.79). As expected, the attitude factor was positively associated with participation in concurrent partnerships, whether assessed by self-report (r = 0.298, p<0.0001) or deduced from dates of recent sexual partnerships (r = 0.298, p<0.0001). The factor was also positively associated with alcohol (r = 0.216, p<0.0001) and drug use (r = 0.225, p<0.0001) and negatively associated with increasing age (r = -0.088, p- = 0.02) and female gender (r = -0.232, p<0.0001). Factor analyses repeated in the second random pre-campaign subsample and post-campaign sample confirmed these results.

**Conclusion:**

A vignette-based scale may be an effective measure of key attitudes related to concurrency and potentially a useful tool to evaluate interventions addressing this network pattern.

## Introduction

African Americans comprised only 13% of the total US population in 2014 [1] yet accounted for almost two-thirds (64%) of persons diagnosed with heterosexually transmitted HIV infection [2]. Concurrent sexual partnerships, sexual partnerships that overlap in time, can increase sexual network connectivity and therefore, the likelihood of HIV transmission [3–6]. Social and economic forces promote a higher prevalence of concurrent partnerships among African Americans [7–10], especially in the Southern US, where rates of other sexually transmitted infections and heterosexually transmitted HIV are high [11]. Although successful control of the U.S. HIV epidemic will ultimately require modifying larger social and structural forces, behavioral interventions are urgently needed to help change sexual network patterns, such as concurrency, that increase HIV transmission.

Little published research documents psychosocial factors that predict participation in concurrency. Several leading theories of behavioral prediction and change, including Fishbein’s integrative model of behavior, [12,13] hold that attitudes, norms, and self-efficacy are primary determinants of intention to perform a behavior [14–16]. Thus, attitudes toward initiation or continuation of a concurrent partnership are a natural target for predicting and influencing behavior. However, the measurement of attitudes towards sexual concurrency has received minimal attention [17]. The context and motivations for engaging in sexual concurrency are complicated and existing scales to measure attitudes toward other sexual behaviors may not be directly adaptable to this network pattern. Qualitative research has identified a variety of motivations for and situations in which concurrency occurs, including transitions between relationships, reactive concurrency (in which one partner has concurrent partnerships in response to the other partner’s concurrency), compensatory concurrency that occurs because of perceived deficits of a partner, separational concurrency during physical separation due, for example, to incarceration, open partnerships, co-parenting, and survival sex [18,19]. Accurate measurement of attitudes toward concurrency may require an instrument that incorporates the nuances of these specific situations.

In this paper we present the development of a scale to measure changes in attitudes toward concurrency to evaluate the effectiveness of a radio campaign to discourage concurrency among young, heterosexual African Americans in Eastern North Carolina, an area with elevated concurrency and HIV prevalence [20,21]. A companion paper reports the results of the campaign (in preparation).

## Methods

### Overall Study Design

Data for this analysis were collected in conjunction with an eight-month radio campaign in Eastern North Carolina (NC) that aimed to 1) inform young, heterosexual African Americans about the association between concurrent sexual partnerships and HIV dissemination in the community and 2) decrease acceptability of concurrency [20]. Computer-assisted telephone interviews (CATI) were conducted with independent samples of people living in 6 largely rural counties, immediately before and after the campaign, to assess changes in behavioral beliefs, attitudes, normative beliefs, and self-efficacy in relation to concurrency and participation in concurrent partnerships. For both the pre- and post-campaign surveys, we used an electronic white page sampling frame with embedded listings of age and race [22,23] and drew a random sample of 18 to 34 year-old African American men and women that could be contacted via landline telephones, with stratification by county to ensure the samples were proportional to population size. Respondents were eligible to participate in surveys if they were not institutionalized, spoke English, resided in one of the six study counties (Edgecombe, Greene, Lenoir, Nash, Pitt and Wilson), and (for the post-campaign survey) had not participated in the pre-campaign survey. All analyses were conducted in SAS version 9.3 (Cary, NC).

### Item Generation and Refinement

We developed an exploratory conceptual model to understand participation in concurrency among African Americans. Using Fishbein’s integrative model of behavior, which holds that attitudes, norms and self-efficacy are primary determinants of intention to perform a specific behavior, [12,13,24] we explored participation in concurrent sexual behavior in our study population. Our model also integrated social constructionist frameworks of gender and power [25,26], constructs that previously have been associated with concurrency [27].

Before initiating the main study, we conducted six focus groups among African American adults in the target age range and counties to identify factors that may promote concurrency in their communities. Focus group participants cited numerous contributors, including low male-to-female ratios, high male incarceration rates, poverty, desire to fulfill sexual and emotional needs that are not met by a main partnership, avoidance of hurt feelings and vulnerability that may accompany a monogamous relationship, retaliation against one’s partner, and having a child with a previous partner [20].

Candidate survey items were adapted from published construct definitions and validated scales for measuring attitudes, norms, self-efficacy, and behavioral beliefs about similar sexual risk behaviors [28–32]. These items used 4-point Likert-type response choices. To ensure that the candidate survey items would be understandable to respondents of the main study survey, preliminary cognitive testing of these items was conducted on a sample of 37 NC African Americans, ages 18 to 45 years, with a CATI-administered instrument. We recruited a small convenience sample of participants for the preliminary cognitive testing by 1) targeted sampling of African American households in NC counties not participating in the main study and 2) inviting nominations from study staff of potential respondents who satisfied the target age and racial categories (to reduce costs for respondent recruitment for preliminary cognitive testing). If more than one eligible respondent resided within the household, young males were oversampled to ensure representation among this important sub-group. Clarity, precision, and general applicability were assessed through a set of follow-up questions asking participants to explain their understanding of the meaning of each item.

Approximately half of respondents who participated in preliminary cognitive testing of the items were men (N = 19, 51%) and most had graduated from high school (N = 33, 89%) and had never been married (N = 23, 62%). Only one respondent had never had sex. Preliminary cognitive testing indicated that the original candidate items measuring attitudes, norms, behavioral beliefs and self-efficacy in relation to concurrent sexual partnerships yielded highly skewed response distributions with limited variability (range of coefficient of variation (measured as 100*[standard deviation/mean]): 33.6–70.0) (Table 1).

**Table 1:** Means, standard deviations, and coefficients of variation for candidate items measuring attitudes, norms, beliefs, and self-efficacy from preliminary cognitive testing (N = 37).

Construct	Item	Mean^d^	Standard Deviation	Coefficient of Variation^e^
	*What is your level of agreement with the following statements?*			
Attitudes	I think it's OK for men to go back and forth between sex partners.^a^	1.22	0.58	48.0
	I think it's OK for women to go back and forth between sex partners.^a^	1.33	0.68	50.7
	I think it's OK if a woman's boyfriend has sex with other people besides her.^a^	1.28	0.78	60.9
	I think it's OK if a man's girlfriend has sex with other people besides her.^a^	1.17	0.61	52.2
	It would be OK for my partner to have sex with someone else.^a^	1.09	0.38	34.8
	It would be OK for me to go back and forth between sex partners.^a^	1.31	0.75	57.4
	It would be OK for my partner to have sex with someone else if, for example, they needed to fulfill different needs.^a^	1.24	0.61	49.1
	It would be OK for me to go back and forth between sex partners if, for example, I needed to fulfill different needs.^a^	1.17	0.51	43.5
	*How confident are you that you can do the following*?			
Self-Efficacy	Stop having sex with your partner if you found out they were having sex with someone else.^b^	1.24	0.55	44.1
	Make sure that you always used a condom if your partner was having sex with other people^b^	1.32	0.75	56.9
	Make sure that you always insisted your partner use condoms if he was having sex with someone else.^b^	1.56	1.09	70.0
	Have only one partner at a time.^b^	1.08	0.36	33.6
	Use a condom with each partner if you had more than one partner.^b^	1.17	0.51	44.1
	Insist that all your partners use condoms if you had more than one partner.^b^	1.24	0.56	45.5
	Ask your partner to use a condom without fear of angering or insulting them.^b^	1.57	1.07	67.8
	*What is your level of agreement with the following statements*?			
Norms	People whose opinions matter to me think I should have only 1 sex partner at a time.^c^	1.44	0.91	62.9
	People whose opinions are important to me think that I should not have sex with someone who has sex with other people besides me.^c^	1.75	1.13	64.6
	People I care about think that I should have only one sex partner at a time.^c^	1.33	0.76	56.7
	My friends think it's okay to sleep with more than one person at a time.^a^	2.08	1.23	58.9
	If the people close to me found out that I was having sex with more than one partner at a time, they would want me to stop.^c^	1.29	0.63	48.6
	In my group of friends it's normal to go back and forth between different sex partners.^a^	1.75	1.05	60.1
	Among people I know, some are in relationships, but also are having sex with other people.^a^	2.50	1.24	49.5
	It is common to be in a relationship, but also have sex with another person.^a^	2.03	1.17	57.5
	My friends think it is normal for me to have sex with other people, even if I am in a relationship.^a^	1.62	1.06	65.6
	When it comes to my sexual relationships, it's important for me to do what people whose opinions matter to me think is right.^a^	2.39	1.32	55.1
	*What is your level of agreement with the following statements*?			
Behavioral Beliefs	When people go back and forth between more than 1 sex partner, it helps spread HIV in the Black community.^c^	1.44	0.77	53.5
	When people go back and forth between sex partners, they spread HIV faster than if they have sex partners one relationship at a time.^c^	1.78	1.03	57.8
	Going back and forth between sex partners helps spread HIV in the Black community.^c^	1.64	0.87	52.9

a Response Scale: strongly agree = 4, somewhat agree = 3, somewhat disagree = 2, strongly disagree = 1

b Response Scale: very confident = 1, somewhat confident = 2, somewhat unconfident = 3, very unconfident = 4

c Response Scale: strongly agree = 1, somewhat agree = 2, somewhat disagree = 3, strongly disagree = 4

d Larger numbers indicate greater acceptance of concurrency

e Coefficients of Variation calculated as 100 x (standard deviation/mean)

Based on the themes emerging from the focus groups and results of the preliminary cognitive testing of the initial candidate items, we developed a set of items incorporating vignettes with realistic scenarios involving concurrency or related behaviors. Each vignette-style item asked the respondent how much s/he agreed that the behavior of fictional characters presented in the scenario was acceptable. We revised items related to self-efficacy by asking respondents to assess the likelihood they would behave in the manner presented in the hypothetical situation. To broaden the potential response distribution and enhance sensitivity to small differences [33], we increased the number of response choices from four to 10 for the attitude and self-efficacy items. Items measuring norms and behavioral beliefs continued to use four response choices.

We conducted cognitive testing of the revised items in a separate convenience sample of 16 African American respondents (6 men and 10 women) between the ages of 18 and 34 years who were recruited and nominated by study staff. To reduce study-related costs, the convenience sample was comprised of individuals known by study staff in the target age and racial categories for the main study. The convenience sample respondents did not live in the study counties and were therefore ensured of exclusion from both the main pre- and post-campaign studies. Responses to the revised items demonstrated greater variability (coefficients of variation range: 21.4–126.3), with vignette-style attitude items and self-efficacy items displaying the most (coefficients of variation range: 66.3–126.3). The revised items were used for the main pre- and post-campaign surveys.

### Factor Analysis

Data collected during the pre- (February through June 2012) and post- (June through November 2013) campaign surveys were used to perform a factor analysis of the revised item set, which served as a content pool from which potential items for the final scale could emerge. Item responses were recoded if necessary so that pro-concurrency responses had higher numerical values. We considered only the revised items with a 10-point response scale (attitude vignette-style items and revised self-efficacy items) in the factor analysis to avoid methods variance due to differences in response formats [34]. The surveys also collected information on respondents’ marital status, sexual behaviors including dates of sexual partnerships in the past year, recent alcohol and drug use, as well as other socio-demographic characteristics.

We randomly divided the pre-campaign survey respondents into two equal-sized groups and performed the same set of analyses on each group, to enable cross-validation of the factor analysis results. To determine the optimal number of factors to retain, we performed an exploratory factor analysis on each half of the pre-campaign sample. The exploratory analysis included an assessment of Cattell’s Scree Plot, Kaiser criterion of Eigenvalues ≥1.0, and the overall interpretability of the resulting factors. The factor analysis used an oblique rotation method that did not require the assumption that the underlying factors were uncorrelated. We retained factors with a primary loading of ≥0.50 and no secondary loading >0.30. Items that loaded poorly across all factors were discarded. Cronbach’s alpha was used to evaluate internal consistency of the items within each individual factor. Factors with low internal reliability (Cronbach’s α<0.7) were not retained. Furthermore, items that factored together but did not make theoretical sense were also discarded. The mean responses of items with a primary loading on the resulting factors were averaged to create composite factor scores. The factor analysis was repeated in the second pre-campaign subsample and in the post-campaign sample. Results from each sample were compared for agreement to confirm the resulting factor solution for our scale assessing concurrency attitudes [34,35].

### Validation

Our conceptual model posits that people who are more accepting of concurrency are more likely to engage in it [14–16]. Thus, a valid scale should be positively correlated with behavioral reports of concurrency and their correlates, and negatively correlated with factors inversely associated with concurrency. To ascertain construct validity, we reviewed existing literature about concurrency and predicted, *a priori*, the direction of the association between the scale and five socio-demographic and behavioral variables measured in the main surveys. Prior research indicates that men, younger adults, unmarried persons, and people with drug and/or heavy alcohol use have greater involvement in concurrency and should accordingly endorse attitudes more accepting of concurrency (i.e., higher scale scores) [7,21,36–38].

We measured concurrency in this population using two methods. First, we provided the definition of concurrency and directly asked respondents if they had participated in this behavior in the past 12 months (self-report). Secondly, we measured concurrency as overlapping sexual partnerships at any point during the past year based on reported dates of first and last sex with the three most recent partners, one of the UNAIDS-recommended definitions [39]. We used direct questioning to characterize binge drinking (report of ≥5 [men] or ≥4 [women] alcoholic drinks on the same occasion on at least 1 day in the past 30 days) and marijuana use (report of smoking marijuana ≥1 time in the last 12 months).

We examined the association between the attitude factor score and known correlates of concurrency using Pearson’s correlation statistic for continuous variables (number of partners in the past 12 months and age) and Spearman’s correlation statistic for categorical variables (sexual concurrency, gender, substance abuse, and marital status). The analysis was conducted separately in the pre- and post-campaign samples. Statistically significant correlation coefficients in the predicted direction were considered as validation that the factor measures attitudes about concurrency.

### Ethics Statement

The University of North Carolina at Chapel Hill Biomedical Institutional Review Board approved all study procedures (IRB #09–2142). Participants in the focus groups, the pilot surveys, and the pre- and post-campaign studies provided written informed consent.

## Results

### Factor Analysis

A total of 1,157 people were surveyed for the main media campaign evaluation. Pre-campaign respondents (N = 678) were predominantly young (46.4% 18–24 years old) and female (59.1%), and most (84.8%) had graduated high school (Table 2). Post-campaign respondents (N = 479) were similar to pre-campaign respondents, with a slightly lower proportion of people who had never been married (69.8% vs. 76.3% pre-campaign).

**Table 2:** Demographic and Risk Characteristics of Pre- and Post-Campaign Respondents.

	Pre-Campaign			Post-Campaign		
	N = 678			N = 479		
	N	%^a^	95% Confidence Interval^a^	N	%^a^	95% Confidence Interval^a^
**Age**						
18–24	256	46.4%	42.1%, 50.7%	190	45.7%	40.8%, 50.6%
25–30	223	29.3%	25.6%, 33.1%	131	23.0%	19.1%, 26.9%
31–35	198	24.3%	20.9%, 27.7%	158	31.4%	27.0%, 35.7%
**Gender**						
Male	237	40.9%	36.5%, 45.2%	177	44.4%	39.5%, 49.3%
Female	441	59.1%	54.8%, 63.5%	302	55.6%	50.7%, 60.5%
**Marital status**						
Married	98	11.2%	8.8%, 13.5%	67	12.0%	9.0%, 15.0%
Cohabitating	61	7.3%	5.2%, 9.4%	68	12.6%	9.4%, 15.8%
Separated, Divorced or Widowed	38	4.8%	3.1%, 6.4%	26	5.4%	3.2%, 7.5%
Never married	479	76.3%	73.0%, 79.7%	317	69.8%	65.4%, 74.1%
**Alcohol & Drug Use**						
Binge drinking past month^b^	203	30.1%	26.2%, 34.1%	141	30.6%	26.0%, 35.2%
Smoked marijuana past year	144	23.2%	19.5%, 26.9%	99	22.9%	18.6%, 27.1%
**Sexual Behavior (past 12 mos)**						
Concurrent partnerships^c^	91	14.7%	11.6%, 17.9%	60	14.4%	10.8%, 18.0%
Self-reported concurrency^d^	102	18.0%	14.4%, 21.6%	62	17.1%	13.0%, 21.2%
**Number of Sex Partners, past year**						
0	47	8.3%	5.7%, 10.8%	40	8.9%	6.0%, 11.7%
1	368	57.2%	52.7%, 61.8%	267	61.4%	56.2%, 66.6%
2	109	21.6%	17.7%, 25.5%	54	15.0%	11.1%, 19.0%
≥3	73	12.9%	9.7%, 16.1%	49	14.7%	10.7%, 18.7%

a Percentages and 95% confidence intervals weighted based on differential sampling and non-response

b Drinking 5+ (men) or 4+ (women) alcoholic drinks on the same occasion on at least 1 day in the past 30 days.

c Overlapping sexual partnerships at any point in the past year inferred from reported dates of sexual partnerships.

d Self-reported concurrency when given the definition of concurrency

The mean response was less than 6 (out of 10) for all revised attitude and self-efficacy items, where 1 denoted the lowest and 10 denoted the highest acceptance of concurrency (Table 3). The item that yielded the mean response least accepting of concurrency was: “How confident are you that you can satisfy your own sexual needs with just one sex partner?” (pre-campaign = 1.70, post-campaign = 1.48). The item with the mean response indicating the most acceptance of concurrency (possibly because while suggestive of concurrency, the scenario does not explicitly indicate its occurrence) was the vignette-styled item that asked participants to rate their acceptance of the following situation: “A woman and the man she used to be with still have feelings for each other and have sex once in a while” (pre-campaign = 5.64, post-campaign = 4.85).

**Table 3:** Survey means and standard deviations for revised items developed from cognitive testing and focus group analysis and included on pre- and post-campaign surveys.

		*Pre-Campaign Random Sample 1 (N = 339)*		*Pre-Campaign Random Sample 2 (N = 339)*		Entire Pre-Campaign (N = 678)		Entire Post-Campaign (N = 479)	
Construct	Items	*Mean*^e^	*Standard Deviation*	*Mean*^e^	*Standard Deviation*	Mean^e^	Standard Deviation	Mean^e^	Standard Deviation
Attitudes (Vignette-Style Items)	**A man has been in a relationship with his girlfriend for three years but she is always working and cannot fulfill his sexual needs. He has sex with an old girlfriend once every couple of months just to meet his needs.**^**a**^	*1*.*74*	*1*.*73*	*1*.*88*	*1*.*96*	1.81	1.85	1.60	1.60
	**A woman does not want to be in a serious relationship so she has a couple of male friends who she sometimes has sex with.** ^**a**^	*3*.*56*	*2*.*89*	*3*.*77*	*2*.*99*	3.66	2.94	2.46	2.42
	**A man doesn’t want to be tied down in a serious relationship so he has a couple of girlfriends that he hooks up with on a regular basis.** ^**a**^	*3*.*76*	*3*.*05*	*3*.*83*	*3*.*08*	3.79	3.06	2.63	2.50
	**A woman and the man she used to be with still have feelings for each other and have sex once in a while.** ^**a**^	*5*.*43*	*3*.*17*	*5*.*86*	*3*.*24*	5.64	3.21	4.85	3.17
	**A man had a serious girlfriend in high school and they have a child together. They are both having sex with other people but sometimes when the man comes to pick up or drop off his child, they have sex.** ^**a**^	*2*.*69*	*2*.*54*	*2*.*83*	*2*.*61*	2.76	2.58	2.04	2.04
	**A man and his girlfriend are living together but have been having relationship problems for months. A week ago a woman he met at a party came on to him really strongly, and they end up having sex at her place.** ^**a**^	*2*.*01*	*2*.*29*	*2*.*03*	*2*.*18*	2.02	2.23	1.69	1.71
	A woman has a child with her boyfriend. She learns that he is also having sex with his ex. She decides to leave him even though he is the father of her child and provides for her financially. ^**a**^^,^^b^	*3*.*45*	*3*.*40*	*3*.*60*	*3*.*43*	3.52	3.41	3.30	3.30
	A man is in a relationship with a woman but they do not have sex very often. Even though the man would like to have sex much more often, he never tries to find another sex partner. ^**a**^^,^^b^	*2*.*54*	*2*.*79*	*2*.*31*	*2*.*64*	2.42	2.72	2.23	2.59
		*Pre-Campaign Random Sample 1 (N = 339)*		*Pre-Campaign Random Sample 2 (N = 339)*		Entire Pre-Campaign (N = 678)		Entire Post-Campaign (N = 479)	
Construct	Items	*Mean*^e^	*Standard Deviation*	*Mean*^e^	*Standard Deviation*	Mean^e^	Standard Deviation	Mean^e^	Standard Deviation
Self-Efficacy	You find out that your [girlfriend/boyfriend] of three years has been having sex with their ex. How likely is it that you that you would end your relationship? ^b^^,^^c^	*1*.*94*	*2*.*11*	*1*.*96*	*2*.*29*	1.95	2.20	1.75	1.96
	You think your [girlfriend/boyfriend] may be having sex with someone else. How likely is it that you would [ask him to] use a condom when the two of you next have sex? ^b^^,^^c^	*1*.*73*	*2*.*06*	*1*.*75*	*2*.*04*	1.74	2.05	1.92	2.34
	How confident are you that you can satisfy your own sexual needs with just one sex partner? ^b^^,^^c^	*1*.*62*	*1*.*89*	*1*.*77*	*1*.*99*	1.70	1.94	1.48	1.48
	You and your boyfriend/girlfriend of three years have a child together. You discover they are having sex with someone else. How confident are you that you can end the relationship? ^b^^,^^c^	*2*.*49*	*2*.*39*	*2*.*29*	*2*.*29*	2.39	2.34	2.26	2.14
Norms	When it comes to my sexual relationships, it’s important for me to do what my friends think best. ^d^	*1*.*15*	*0*.*63*	*1*.*13*	*0*.*49*	1.14	0.56	1.11	0.40
	In my group of friends it's normal to have more than 1 sexual relationship at a time. ^d^	*1*.*60*	*1*.*09*	*1*.*65*	*1*.*08*	1.62	1.09	1.55	0.96
	Among people I know, some are in relationships but are also having sex with other people. ^d^	*2*.*52*	*1*.*23*	*2*.*65*	*1*.*29*	2.58	1.26	2.67	1.29
	My friends think it’s okay to sleep with more than one person at a time. ^d^	*1*.*97*	*1*.*15*	*2*.*02*	*1*.*23*	1.99	1.19	2.13	1.25
	It is common to be in a relationship but also have sex with another person. ^d^	*2*.*13*	*1*.*22*	*2*.*04*	*1*.*22*	2.08	1.22	2.02	1.20
Behavioral Beliefs	Concurrency helps spread HIV. ^b^^,^^d^	*1*.*04*	*0*.*21*	*1*.*07*	*0*.*26*	1.06	0.23	1.20	0.57
	Concurrency is a problem in our community. ^b^^,^^d^	*1*.*07*	*0*.*25*	*1*.*13*	*0*.*73*	1.10	0.54	1.27	0.81
	Concurrency has a negative impact on our children. ^b^^,^^d^	*1*.*08*	*0*.*27*	*1*.*10*	*0*.*30*	1.09	0.29	1.33	0.76

a Response Scale: On a scale of 1 to 10, with 1 being not at all okay and 10 being completely okay

b Scale reversed so larger number indicates a “pro-concurrency” response

c Response Scale: On a scale of 1 to 10, with 1 being not at all likely and 10 being very likely

d Response Scale: strongly agree = 1, somewhat agree = 2, somewhat disagree = 3, strongly disagree = 4

e Larger number indicated pro-concurrency response

Bolded items included in final factor

**Fig 1:**
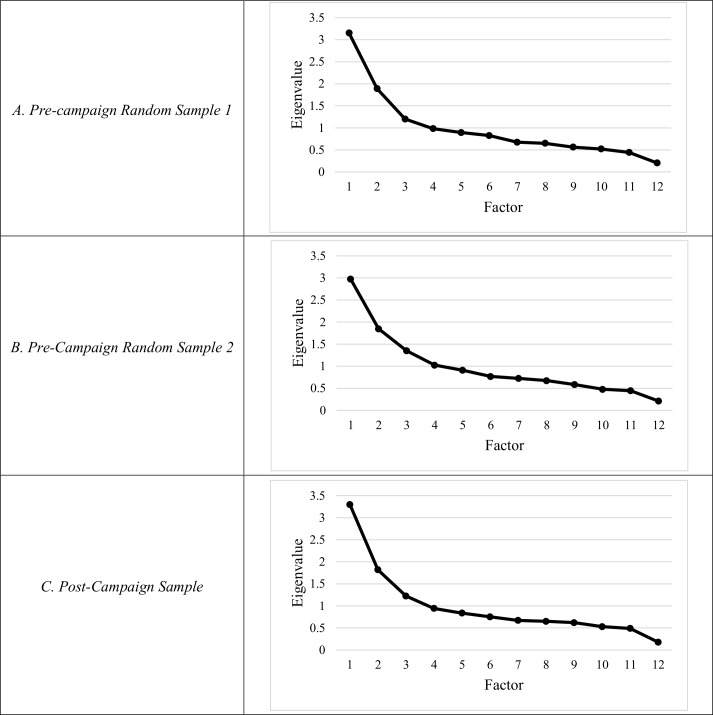
Cattell’s Scree Plot. Cattell’s Scree Plot of the raw eigenvalues for the (A) first pre-campaign random sample, (B) second pre-campaign random sample and (C) the post-campaign sample.

In the first random pre-campaign subsample, three factors emerged with Eigenvalues greater than 1.00 (3.08; 1.84; 1.24) among the revised attitude and self-efficacy items; these three factors accounted for 53.6% of the total item variance. Upon visual inspection, the scree plot also suggested that three factors should be retained in the factor analysis (Fig 1A).

Examination of the factor loading scores from the factor analysis revealed that most of the revised items loaded onto only one factor. Moreover, four revised items displayed evidence of loading across two different factors. Cronbach’s alpha supported internal reliability only for Factor 1 (α = 0.82); reliabilities for Factor 2 and Factor 3 were 0.53 and 0.50, respectively. Even after removing revised items that cross-loaded across more than one factor, reliability estimates for factors 2 and 3 were low. The revised items that loaded onto Factor 1 were all vignettes and clearly related to attitudes toward concurrency. The interpretation of Factors 2 and 3 was less straightforward, with revised items associated with both self-efficacy and attitude loading onto both factors. The resulting low internal consistency and lack of interpretability prompted us to remove Factors 2 and 3 from further consideration.

After we restricted the analysis to only one factor, loadings for six revised items, all vignettes, were high (>0.50). The remaining six revised items had low loadings (<0.40) and were removed from further consideration. We re-analyzed the reduced vignette-style item pool, extracting only a single factor [Table 4]; this factor accounted for 51.6% of the total variance and had acceptable reliability (Cronbach’s α = 0.79). Very similar results were obtained from the second pre-campaign subsample (48.4% of total item variance, Cronbach’s α = 0.79) as well as from the entire post-campaign sample (51.1% of variance, Cronbach’s α = 0.80) (Fig 1B and 1C and Table 4).

**Table 4:** Factor Analysis of vignette-style items included in final factor for measuring attitudes toward concurrency.

	Factor 1 Loading Scores		
Item	Pre-Campaign Random Sample 1	Pre-Campaign Random Sample 2	Post-Campaign Sample
A man doesn’t want to be tied down in a serious relationship so he has a couple of girlfriends that he hooks up with on a regular basis.	0.803	0.854	0.852
A woman does not want to be in a serious relationship so she has a couple of male friends who she sometimes has sex with.	0.799	0.787	0.793
A man had a serious girlfriend in high school and they have a child together. They are both having sex with other people but sometimes when the man comes to pick up or drop off his child, they have sex.	0.764	0.723	0.723
A woman and the man she used to be with still have feelings for each other and have sex once in a while.	0.722	0.710	0.626
A man and his girlfriend are living together but have been having relationship problems for months. A week ago a woman he met at a party came on to him really strongly, and they end up having sex at her place.	0.598	0.554	0.617
A man has been in a relationship with his girlfriend for three years but she is always working and cannot fulfill his sexual needs. He has sex with an old girlfriend once every couple of months just to meet his needs.	0.566	0.512	0.644
Factor Score: Mean (Standard Deviation)	3.20 (1.89)	3.37 (1.90)	2.54 (1.61)
Cronbach's alpha	0.79	0.79	0.80
Total Item Variance	51.6%	48.4%	51.1%

### Validation

In the pre-campaign sample, our hypothesized attitude factor displayed a statistically significant positive association with having participated in concurrent partnerships, whether assessed by self-report (r = 0.298, p<0.0001) or deduced from dates of recent sexual partnerships (r = 0.298, p<0.0001). In addition, the factor score was positively correlated with reported number of sexual partners in the past year (r = 0.350, p<0.0001), a variable that is also strongly correlated with involvement in concurrency. Similar associations were observed in the post-campaign sample (Table 5).

**Table 5:** Validation of Factor: Associations between the attitude factor score and known correlates of concurrency.

	Predicted Association(+ positive;–negative)	Rationale	Pre-Campaign Correlation (N = 678)		Post-Campaign Correlation(N = 479)	
			r^a^	p-value	r^a^	p-value
Sexual Behavior Variables						
Concurrency in past 12 months (based on dates)	+	People engaging in concurrent partnerships will have attitudes favorable toward concurrency.	0.298	<0.0001	0.277	<0.0001
Self-reported concurrency in past 12 months (when given definition)	+	People engaging in concurrent partnerships will have attitudes favorable toward concurrency.	0.298	<0.0001	0.325	<0.0001
Number of sex partners past year	+	People with more sex partners in the past year will be more likely to engage in concurrent partnerships [36] and will therefore have attitudes favorable toward concurrency.	0.350	<0.0001	0.445	<0.0001
Demographic Variables						
Binge drinking past month^b^	+	The prevalence of concurrency is higher among people who binge drink [37]. Therefore respondents reporting binge drinking will be more likely to have attitudes favorable toward concurrency.	0.216	<0.0001	0.178	0.0001
Smoked marijuana in past year	+	The prevalence of concurrency is higher among people who use marijuana [37]. Therefore respondents reporting marijuana use will be more likely to have attitudes favorable toward concurrency.	0.225	<0.0001	0.316	<0.0001
Age	–	The prevalence of concurrency is lower among older people [36,37]. Therefore older respondents will be less likely to have attitudes favorable toward concurrency.	-0.088	0.02	-0.116	0.01
Gender	–	The prevalence of concurrency is lower among females than males [7,21,38]. Therefore female respondents will be less likely to have attitudes favorable toward concurrency.	-0.232	<0.0001	-0.274	<0.0001
Marital Status	–	The prevalence of concurrency is lower among people who are married [7,21,36–38]. Therefore married respondents will be less likely to have attitudes favorable toward concurrency.	-0.14	0.7	-0.087	0.06

a. Pearson’s correlation statistic was used with continuous variables (number of partners in the past 12 months and age) and Spearman’s correlation statistic was used with categorical variables (sexual concurrency, gender, substance abuse, and marital status).

b. Drinking 5+ (men) or 4+ (women) alcoholic drinks on the same occasion on at least 1 day in the past 30 days.

As predicted *a priori*, attitude factor scores were positively associated with binge drinking and marijuana use in both the pre- (binge drinking r = 0.216, p<0.0001; marijuana use r = 0.225, p<0.0001) and the post-campaign samples (binge drinking r = 0.178, p = 0.0001; marijuana use r = 0.316, p<0.0001). Similarly, the data supported our expectations that women would display attitudes indicating lower acceptance of concurrency than did men (pre-campaign r = -0.232, p<0.0001; post-campaign r = -0.274, p<0.0001) and that older respondents would have attitudes less accepting of concurrency than younger respondents (pre-campaign r = -0.088, p = 0.02; post-campaign r = -0.116, p = 0.01). However, contrary to our expectations, we did not observe married respondents to have lower acceptance of concurrency (pre-campaign r = -0.14 p = 0. 7; post-campaign r = -0.087 p = 0.06) [Table 5].

## Discussion

In this paper we report the development and evaluation of a scale for measuring attitudes toward concurrent sexual partnerships among young African American adults in the general population in Eastern North Carolina. Items adapted from previously published scales assessing attitudes about sexual behaviors had highly-skewed response distributions with insufficient variability for assessing concurrency-related attitudes in this population. Using feedback about these items and qualitative research, we developed vignette-style items that exhibited greater variability in responses. Factor analysis of the vignette-style items yielded a single concurrency attitude factor with acceptable internal consistency. The single factor was associated with concurrency and known correlates of concurrency in the predicted directions, providing construct validity. Thus, we believe that these results suggest that this scale may accurately assess young African Americans’ attitudes toward concurrency in a variety of realistic situations.

Behavioral theory regards attitudes as a critical construct for predicting behavioral intention and behavior itself [14–16,40]. However, there is little published research on methods for measuring attitudes toward sexual behavior other than condom use [17,28–32]. The candidate set of items used during our cognitive assessment were adapted from definitions of psychosocial constructs and previously validated scales used to assess attitudes, norms, and self-efficacy toward related sexual risk behaviors [28–32]. However, the distributions of responses to these items in cognitive testing had very low variability.

Results of focus group discussions helped us develop a pool of vignette-style items that respondents understood and were willing to endorse. The new items referred to fictional characters in common situations. Unlike the items adapted from existing scales, the vignettes may have encouraged respondents to think of familiar real-life situations, possibly even reminding them of people they knew. Respondents were still asked to provide their personal opinions of stigmatized behaviors but were not directly evaluating their *own* behavior. Given the sensitivity of concurrency, a third-person perspective may diminish respondents’ fear of socially undesirable responses [41]. The respondent can countenance others’ potentially stigmatized behaviors without necessarily endorsing them or suggesting that the respondent would engage in them. This greater distance from the behavior may allow respondents to be more comfortable in answering honestly, as their responses reflect less on them as individuals. However, it is possible that individuals hold differing views for themselves and for people more generally, which would lower validity when the variable of interest is an assessment of the respondent her/himself. Despite this disadvantage, the vignette-based items appeared to be valid in our analyses. In addition, 10-point response scales may have been more sensitive to nuances of feeling than 4-point response scales.

Although vignettes have been used to assess psychosocial constructs related to sexual health, [42–45] we are not aware of a previous instance in which a factor analysis approach empirically validated a scale of vignette-style items. Exploratory factor analysis provides a more rigorous replication test than confirmatory factor analysis [35]. Therefore, we chose to replicate the exploratory factor analysis conducted on the first half of the pre-campaign sample on a second sample to provide confirmation or cross-validation of results. The same factor with the same set of vignette-style items and similar reliability estimates emerged in each sample, supporting our conclusion of a one-factor solution. The revised self-efficacy items used in this analysis resulted in a factor with low internal consistency, suggesting that further refinement is necessary to measure this construct in this population. We were unable to perform test-retest assessment in the same sample because this study consisted of two independent cross-sectional samples. We believe that internal consistency may more directly address the reliability of the instrument than test-retest methods, which are susceptible to 1) confounding of true reliability with stability in the construct over time and 2) data contamination due to respondents’ level of motivation at the initial and subsequent testing time points (i.e. deliberative effects) [46]. However, we recommend that future users of this scale consider test-retest assessments to examine temporal stability within their populations.

Formal validation provided further evidence that the identified factor measures attitudes about concurrency. Our adaptation of Fishbein’s integrative model of behavior theorizes that certain socio-demographic traits and behavioral beliefs are associated with individual concurrency attitudes, which in turn influence individual behavior. We observed associations in the predicted direction between the factor score and known correlates of concurrency (e.g., male sex, youth, and substance abuse) [7,21,36–38], supporting validation of the scale. However, we did not observe an association between pro-concurrency attitudes on this scale and unmarried status, which may be partly due to the relatively small numbers of married respondents in both the pre-campaign (11.2%) and post-campaign (12.0%) samples. Although some of the correlations with validation criteria were modest, it is worth noting that the latter were not measures of closely-related constructs but in some cases patterns of behavior (e.g., alcohol and marijuana consumption) that have multiple determinants or demographic factors (e.g., gender, age) that influence attitudes. Although we believe that these are relevant to attitudes toward concurrency, their relationship to those attitudes is somewhat indirect. Consequently, we regard the fact that the associations observed were modest as neither surprising nor discouraging. Rather, we believe that they constitute encouraging preliminary evidence for the validity of the concurrency attitude factor in this and similar populations. As with any new instrument, periodic review of this scale will be needed to establish its validity among African Americans from different parts of the country and for persons from other racial/ethnic backgrounds.

Surveys were conducted over the telephone, protecting respondents’ privacy in providing information about their attitudes and sexual behaviors and reducing potential social desirability bias in their responses. While a growing number of households rely only on cell phone service, the portability of cell phone numbers makes it difficult to target a specific geographic area and would have required a substantial increase in our research budget. Although respondents lived in a household with a landline, most respondents owned a cellular phone (84%, data not show), similar to levels of cell phone ownership reported by other sources [47]. Comparison to the American Community Survey indicated that our sample included adequate representation by gender, age, and marital status for this geographic region (Results paper, in preparation).

The scale developed in this analysis has promise for measuring attitudes about acceptability of concurrent sexual partnerships. By incorporating situations known to prompt sexual concurrency in this and other settings, the vignette-style items appeared to sensitively and reliably measure variation in attitudes about concurrency among young African Americans in the general population of Eastern North Carolina. Measuring attitudes toward concurrency may serve as an indicator of sexual risk behavior, and a measurable outcome in the evaluation of behavioral HIV prevention initiatives.
